# PCRFed: personalized federated learning with contrastive representation for non-independently and identically distributed medical image segmentation

**DOI:** 10.1186/s42492-025-00191-0

**Published:** 2025-03-28

**Authors:** Shengyuan Liu, Ruofan Zhang, Mengjie Fang, Hailin Li, Tianwang Xun, Zipei Wang, Wenting Shang, Jie Tian, Di Dong

**Affiliations:** 1 Department of Electronic Engineering, The Chinese University of Hong Kong, Hong Kong, 999077 China; 2 School of Artificial Intelligence, University of Chinese Academy of Sciences, Beijing, 100049 China; 3 Key Laboratory of Molecular Imaging, Institute of Automation, Chinese Academy of Sciences, Beijing, 100190 China; 4 Beijing Advanced Innovation Center for Big Data-Based Precision Medicine, School of Engineering Medicine, Beihang University, Beijing, 100191 China; 5 The Artificial Intelligence and Intelligent Operation Center, China Mobile Research Institute, Beijing, 100053 China

**Keywords:** Data privacy preservation, Client-specific adaptation, Distributed model regularization

## Abstract

Federated learning (FL) has shown great potential in addressing data privacy issues in medical image analysis. However, varying data distributions across different sites can create challenges in aggregating client models and achieving good global model performance. In this study, we propose a novel personalized contrastive representation FL framework, named PCRFed, which leverages contrastive representation learning to address the non-independent and identically distributed (non-IID) challenge and dynamically adjusts the distance between local clients and the global model to improve each client’s performance without incurring additional communication costs. The proposed weighted model-contrastive loss provides additional regularization for local models, optimizing their respective distributions while effectively utilizing information from all clients to mitigate performance challenges caused by insufficient local data. The PCRFed approach was evaluated on two non-IID medical image segmentation datasets, and the results show that it outperforms several state-of-the-art FL frameworks, achieving higher single-client performance while ensuring privacy preservation and minimal communication costs. Our PCRFed framework can be adapted to various encoder-decoder segmentation network architectures and holds significant potential for advancing the use of FL in real-world medical applications. Based on a multi-center dataset, our framework demonstrates superior overall performance and higher single-client performance, achieving a 2.63% increase in the average Dice score for prostate segmentation.

## Introduction

As data-driven approaches, deep-learning-based medical image segmentation methods have demonstrated exceptional performance in medical image analysis [1–5]. These advanced techniques are now applied in clinical practice to assist physicians in making informed decisions [6, 7]. Collaborative training using data from multiple medical sites is essential for building accurate and robust deep networks for medical image segmentation. However, facilitating data communication across multiple sites is generally infeasible due to patient privacy protection concerns.

**Fig. 1:**
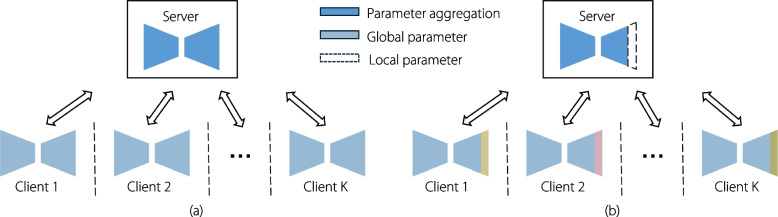
Comparison of traditional and PFL frameworks: traditional FL algorithms (**a**) aggregate all client models to generate a single global model. In contrast, PFL algorithms (**b**) retain certain local parameters while leveraging the global model, allowing each client to make personalized adjustments based on the unique characteristics of their own data

Federated learning (FL) has recently emerged as a promising privacy-preserving approach (Fig. 1a), enabling the training of models on distributed datasets while keeping data local [8, 9]. In healthcare, FL can improve patient care by enabling the development of more accurate, individualized models for medical imaging without compromising privacy. FL allows healthcare organizations to collaborate without sharing sensitive data, thereby enhancing access to advanced artificial intelligence tools and reducing healthcare disparities. Personalized FL (PFL) offers a pathway to creating robust, privacy-preserving models that improve the accuracy and efficiency of healthcare delivery, leading to enhanced clinical decision-making and better patient outcomes, particularly in data-scarce or resource-constrained environments. In a typical FL paradigm, such as FedAvg [10], each local client, such as a medical site or hospital, trains its own model using private data [11]. A global server periodically aggregates the model parameters from multiple clients and distributes the updated model back to them. FL has gained recognition as a crucial domain in machine learning, attracting considerable attention from researchers [12, 13].

A key and common challenge in FL is that the data distributions of different parties are typically non-independent and identically distributed (non-IID) [14, 15]. In real-world medical FL scenarios, inter-client data heterogeneity is significantly more severe than in natural images. Factors such as differences in imaging devices, scanners, protocols, and variability in patient populations can profoundly influence the distribution of imaging data, ultimately degrading FL performance. Numerous studies have been conducted to address the non-IID issue, which can be classified into two main categories: improving local training at each client and enhancing aggregation at the server.

Regarding research focused on improving local training, FedProx [16] introduced a proximal term into the objective function during local training to constrain the distance between local and global models. As a result, the local update is directly regulated by the $L_{2}$ regularization term. FedBN [17] mitigates data distribution shifts by keeping batch normalization (BN) layers local, while MOON [18] enhances consistency between local and global models through contrastive learning on client-side latent feature representations. For studies aimed at improving the aggregation process, FedNova [19] normalizes local updates based on each client’s local epochs before integrating them into the global model. Additionally, HarmoFL [20] and the framework proposed by Chen et al. [21] leverage a Fourier transform to map client models into the frequency domain, averaging low-frequency parameters while preserving high-frequency components unique to each client. Our study primarily focuses on improving client training and selecting optimal parameters for aggregation before sending them to the server. As a result, our research remains independent of the second category and has the potential to be integrated with these aggregation-based methods.

Most existing literature focuses on developing a single global model that exhibits robustness and generalization across multiple clients. However, due to data heterogeneity and quantity skew, a single global model may fail to achieve optimal performance at all local sites. Variations in data distributions across sites and imbalances in data quantity can lead to biased model training and poor generalization. To address this, we employ PFL to enhance client-specific performance by partitioning the local model into global and personalized components (Fig. 1b). This study introduces a PFL framework that integrates contrastive representation learning [22–24], a self-supervised technique that optimizes data representations by maximizing similarity between positive sample pairs while minimizing similarity between negative pairs through a contrastive loss function. This approach enables adaptive adjustment of the distance between local client models and the global model.

The main contributions of this study are as follows:

- We develop a novel PFL framework named PCRFed for medical image segmentation to improve the performance of each local client without extra communication costs. The proposed framework is adaptable to different Encoder-Decoder segmentation network architectures.
- We propose a weighted model-contrastive loss to provide additional regularization for local models. This enables local models to optimize towards their own distribution while effectively utilizing the information provided by all clients to mitigate the challenges caused by insufficient local data.
- We conduct extensive experiments on two medical image segmentation datasets, namely, prostate magnetic resonance imaging (MRI) and cervical cancer computed tomography (CT) image segmentation datasets. Our framework achieves superior performance compared with state-of-the-art methods, especially where the sample distributions and quantities are imbalanced across different clients.

### Related work

#### Medical image segmentation

Medical image segmentation is essential for computer-aided diagnosis and quantitative analysis of various medical conditions. It involves partitioning an image into multiple regions, each representing a different anatomical structure or pathological entity. However, medical image segmentation poses several challenges, including the complexity and variability of anatomical structures, noise and artifacts, and limited annotated data. Deep learning has demonstrated remarkable success in medical image segmentation [25, 26]. U-Net [27], a convolutional neural network with an encoder-decoder structure and skip connections, is one of the most widely used architectures. Its ability to capture both local and global context information enables accurate segmentation with relatively few parameters. To address the increasing scale and complexity of medical tasks, various U-Net extensions, such as AttentionUNet [28] and HDenseUNet [29], have been developed. Recently, Transformer-based methods have gained attention in medical image segmentation due to their ability to model long-range dependencies and global representations [30, 31]. Hybrid approaches, such as TransUNet [32] and SwinUNet [33], integrate Transformer modules into the U-Net framework, enhancing feature extraction and fusion capabilities. The encoder-decoder structure remains a dominant and effective approach due to its ability to extract and combine multiscale features while generating precise pixel-wise predictions. In our proposed PFL framework, we leverage latent feature representations from the encoder to enhance agreement between local and global models, making the framework adaptable to various network architectures.

#### PFL

PFL aims to learn personalized local models for each client in order to adapt the global model to each client’s local data distribution. This type of FL focuses on the individual needs of each client, allowing the creation of unique models for each individual’s local dataset. By adapting the global model to the specific data of each client, the models can become more accurate and efficient in predicting outcomes. Recent methods split the network architecture into shared and personalized layers, reducing the number of communication parameters uploaded to the server [28, 34]. Partial parameters are sent to the global server for updating (global part), whereas personalized parameters remain at the local site and are not aggregated by the server. For example, FedRep [35] employs a shared feature extractor and personalized classification head for each client. FedBN [17] maintains the BN layers locally without uploading them to a central server. Chen et al. [36] proposed a progressive Fourier aggregation module that converted client parameters into the frequency domain. This method then extracts low-frequency components as global components while leaving the high-frequency components personalized, thus ensuring the retention of client-specific knowledge. However, determining which aspects of model parameters are shared across clients and which to personalize remains a challenging task. To address this issue, we presented a unified PFL framework and conducted extensive experiments to determine its ideal configuration.

#### Contrastive learning

Contrastive learning [22, 23] has emerged as a self-supervised technique that aims to learn informative and meaningful representations of data. It leverages the similarities and differences between instances to learn these representations by maximizing the agreement between positive pairs of samples and minimizing the agreement between negative pairs. This is achieved using a loss function known as contrastive loss, which measures the similarity of the representations of different instances in a dataset or batch. SimCLR [23] is a simple yet effective framework for contrastive learning. It applies a normalized temperature-scaled cross-entropy (NT-Xent) loss function to compare the representations of two augmented views of the same image, both against each other and against the representations of other images in the batch. Given a minibatch containing *N* images, SimCLR generates 2*N* augmented views. The NT-Xent loss for a pair of representations $z_{i}$ and $z_{j}$ corresponding to image *z* can be defined as follows:

1 $$\begin{aligned} l_{i, j}=-\log \frac{\exp \left( sim \left( z_{i}, z_{j}\right) / \tau \right) }{\sum _{k=1}^{2 N} \mathbbm {1}_{[k \ne i]} \exp \left( sim \left( z_{i}, z_{k}\right) / \tau \right) } \end{aligned}$$

where $sim(a,b) = a^{T}b/\Vert a\Vert \Vert b \Vert$ is a similarity function and $\tau$ is a temperature hyperparameter. Recent studies [18] combined contrastive learning with FL to improve consistency between local and global models. In this study, we propose a weighted model-contrastive loss based on NT-Xent loss in SimCLR and utilize latent feature representation in medical image segmentation tasks.

## Methods

**Fig. 2:**
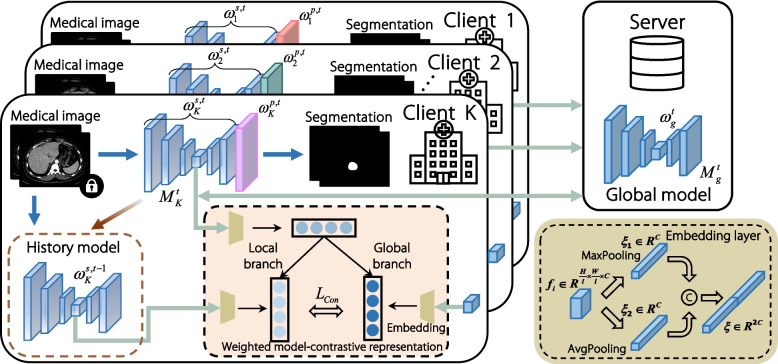
Overview of our proposed PFL framework with contrastive representation for medical image segmentation

In this study, we designed a PFL framework with contrastive representation for medical image segmentation. This section introduces the formulation of personalized federated medical image segmentation and network architecture, followed by an explanation of the design rationale and implementation. A comprehensive discussion of the datasets, implementation, and experimental methodology is presented. Figure 2 illustrates the overall pipeline of the proposed approach.

### Preliminaries

We denote ($\mathcal {X}$ and $\mathcal {Y}$) as the joint image and label space of the segmentation task, respectively. There are *K* clients $\mathcal {P} = \{P_{1}, P_{2}, \dots , P_{K}\}$, and each client contains $n_{k}$ data and label pairs $P_{k} = \{ ( x_{k}^{i}, y_{k}^{i}) \}_{i=1}^{n_{k}}$. The FL paradigm includes communication between *K* clients and the central server. In every communication round *t*, the central server sends parameters $\omega _{g}^{t}$ to each client $P_k$, which then updates its local model $M_{k}^{t}$ for *E* epochs. After receiving the updated models from all the clients, the central server aggregates them to update the global model $M_{g}^{t}$. For the aggregation operation, this study focuses on the most popular federated averaging algorithm, FedAvg [10]. It aggregates local parameters by weighing them proportionally to the size of each local dataset, which updates the global model.

2 $$\begin{aligned} \omega _{g}^{t+1} = \sum _{k=1}^{K} \frac{n_{k}}{N} \omega _{k}^{t} \end{aligned}$$

where *N* is the total number of samples included for all clients.

Under IID conditions, the global optimum $\omega _{g}^{*}$ is close to the local optima $\omega _{1}^{*}$ and $\omega _{2}^{*}$. However, in non-IID scenarios, because $\omega _{g}^{*}$ is far from $\omega _{k}^{*}$, $\omega _{k}^{t+1}$ may also be far from $\omega _{g}^{*}$. Developing an effective FL algorithm to design a single shared model in a non-IID setting is a significant challenge. Consequently, we introduce our PFL framework to address non-IID data.

### Network architecture

**Figure:**
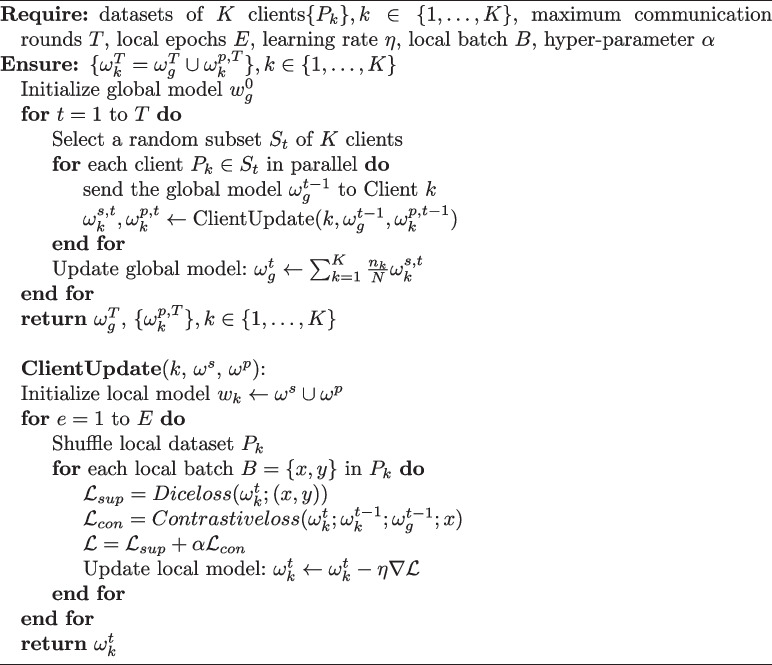
**Algorithm 1** PCRFed framework

The developed PCRFed framework is applicable to most encoder-decoder deep neural networks. The primary objective of this study is to validate the effectiveness of the proposed PFL algorithm. For this purpose, we employ the ubiquitous and fundamental UNet [27] architecture as the base network for our clients, where the encoder extracts latent features and the decoder generates a pixel-wise mask. Instead of learning a single shared model, our proposed PCRFed framework is designed to learn *K* unique models $\{M_{i}\}_{i=1}^{K}$, each tailored to a specific local client, to obtain optimal information for each client. As shown in Fig. 2, we divide the local client model into two components: a global shared part, denoted as $\omega _{k}^{s}$, and a personalized part, represented by $\omega _{k}^{p}$. Consequently, during the communication round *t*, we have $\omega _{k}^{t} = \omega _{k}^{s,t} \cup \omega _{k}^{p,t}$.

During the client update step, the local loss comprises two components. The first component is a supervised loss, denoted by $\mathcal {L}_{sup}$, and the second is the proposed weighted model-contrastive loss, represented by $\mathcal {L}_{con}$. For each client *k* with $n_{k}$ samples (*x*, *y*), the supervised loss is a weighted combination of the Dice loss and binary cross-entropy (BCE) loss. This loss function supervises the model at the pixel level and measures the difference between the predicted probability distribution and the ground-truth segmentation mask.

3 $$\begin{aligned} \mathcal {L}_{sup} = \mathcal {L}_{Dice} + \mu \mathcal {L}_{BCE} \end{aligned}$$

4 $$\begin{aligned} \mathcal {L}_{Dice} =\frac{1}{n_{k}} \sum _{i=1}^{n_{k}} (1 - 2 \times \frac{ | y_i * \hat{y}_i|}{|y_{i}| + |\hat{y}_i|}) \end{aligned}$$

5 $$\begin{aligned} \mathcal {L}_{BCE}=-\frac{1}{n_{k}} \sum _{i=1}^{n_{k}} (y_i \log (\hat{y}_i) + (1 - y_i) \log (1 - \hat{y}_i)) \end{aligned}$$

where $y_i$ represents the ground truth segmentation mask and $\hat{y}_i = M^{t}_{k}(x_{i}; \omega _{k}^{t}$) denotes the predicted mask.

### Contrastive representation for a non-IID problem

To address the non-IID issue, we intuitively adjust the optimization direction of the model. An excellent personalized model should fully leverage all data to mitigate local data scarcity while optimizing the distribution for each client. Therefore, we propose a weighted model-contrastive loss to provide additional regularization for the local models.

For every input *x*, we extract the latent representation *R*(*x*) from the bridge component located after the encoder of the UNet-based network. Specifically, the global model representation of *x* is given by $R_{\omega ^{t}_{g}}(x)$, while the local client representation is denoted as $R_{\omega ^{t}_{k}}(x)$. The local client retains the parameters from the previous round to generate the feature representation $R_{\omega ^{t-1}_{k}}(x)$. To enhance the model’s feature representation, we introduce a site embedding layer without adding additional parameters. The structure of this embedding layer is illustrated in Fig. 2. This layer, denoted as $F(\cdot )$, employs both average-pooling and max-pooling operations. By integrating these two pooling techniques, the structure effectively extracts a diverse set of features, capturing both general patterns and the most prominent information within the embedding layer. Given input *x*, the model representations are obtained as $\xi _{g} = F(R_{\omega ^{t}_{g}}(x))$, $\xi = F(R_{\omega ^{t}_{k}}(x))$, and $\xi _{b} = F(R_{\omega ^{t-1}_{k}}(x))$. Similar to NT-Xent loss [23], we define weighted model-contrastive loss as

6 $$\begin{aligned} \mathcal {L}_{con} = -\beta \log \frac{\exp \left( sim \left( \xi , \xi _g\right) / \tau \right) }{\exp \left( sim \left( \xi , \xi _g\right) / \tau \right) + \exp \left( sim \left( \xi , \xi _{b}\right) / \tau \right) } \end{aligned}$$

where $\beta = e^{- \frac{n_{k}}{N}}$ is an adaptive parameter designed to fine-tune the optimization direction of the local models. A larger value was employed for clients with limited sample sizes, effectively leveraging global information to guide the convergence of the model and mitigate overfitting issues caused by data insufficiency.

### Overall loss function

Thus, the overall training loss for each local client is defined as

7 $$\begin{aligned} \mathcal {L} = \mathcal {L}_{sup}(\omega _{k}^{t}; (x,y)) + \alpha \mathcal {L}_{con}(\omega _{k}^{t}; \omega _{k}^{t-1}; \omega _{g}^{t-1}; x) \end{aligned}$$

where $\alpha$ is a hyperparameter that balances the weights of the supervised and contrastive losses.

The overall PCRFed learning algorithm is presented in Algorithm 1. During each local training session, clients utilize the Adam optimizer to update their local models using their own data and subsequently transmit the shared portion of the parameters to aggregate the global model.

### Datasets and implementation

We conducted experiments on two non-IID multiclient datasets for segmentation tasks: prostate segmentation from T2-weighted MRI images [37, 38] and cervical tumor segmentation from CT images.

Prostate MRI images (https://liuquande.github.io/SAML/) were acquired from six public sites using different acquisition protocols and were manually annotated for prostate segmentation. The images had heterogeneous in-plane and through-plane resolutions. All images were preprocessed to ensure a consistent field of view and subsequently resampled to 384 $\times$ 384 pixels in the axial plane. The sample numbers for each site were {421, 384, 468, 175, 261, and 158}. For prostate MRI images, ethical approval was obtained from patients included in the open-source database.

Cervical CT images were obtained from eight different centers, as described in our previous study [39, 40]. We collected 1077 samples with a resolution of 512 $\times$ 512 pixels; detailed information is provided in Table 1. For each patient, a gynecologist with five years of experience selected the slice containing the largest tumor area from the CT images and segmented the tumor region of interest (ROI) using ITK-SNAP software (http://www.itksnap.org/). Another gynecologist with ten years of experience verified all ROIs. CT images from all centers were linearly interpolated to a pixel size of 1 mm $\times$ 1 mm $\times$ 1 mm. Before training, the data were center-cropped to 256 $\times$ 256 to serve as model inputs. This study was approved by the institutional review boards of all eight hospitals listed in Table 1. The institutional review boards of all hospitals waived the requirement for written informed consent.

**Table 1:** Detailed information of the cervical cancer dataset

Index	Site names	Number
1	The Affiliated Hospital of Qingdao University	255
2	Nanfang Hospital	219
3	Jiangmen Central Hospital	197
4	Fourth Hospital of Hebei Medical University	129
5	Daping Hospital of Third Military Medical University	41
6	The First Affiliated Hospital of Zhengzhou University	43
7	Yuhuangding Hospital	76
8	Guizhou Provincial Hospital	73
	Total	1033

**Fig. 3:**
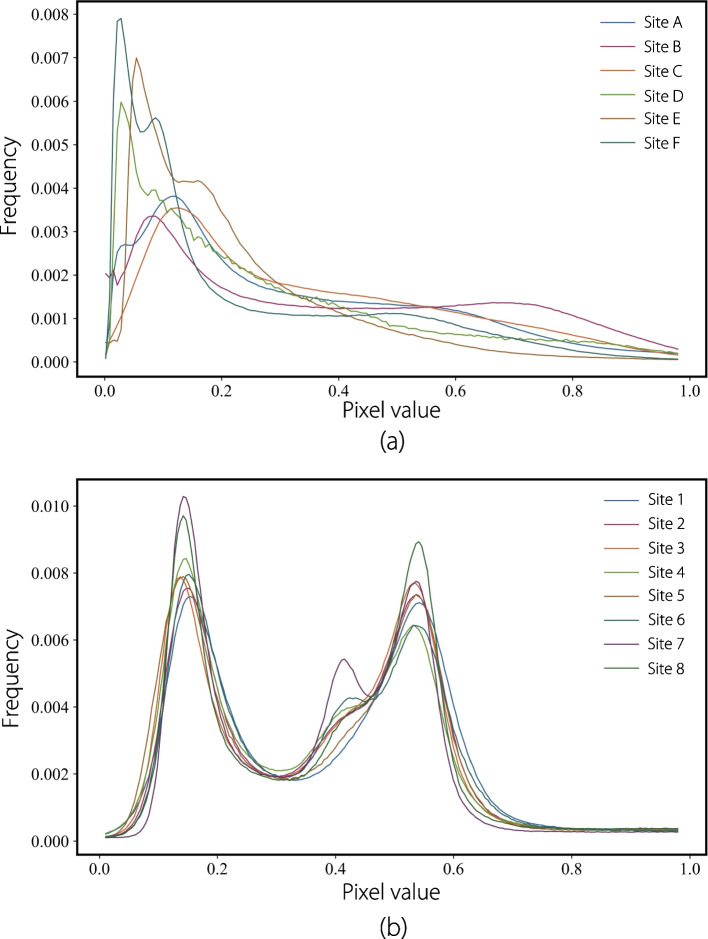
The overall pixel distribution of two segmentation datasets after intensity normalization. **a** The distribution of the prostate MRI dataset; **b** The distribution of the cervical cancer CT dataset

To mitigate intensity variance across different sites, we performed sample normalization to a range of (0, 1) before inputting the images into the network. Figure 3 shows that both datasets exhibited non-IID characteristics in their intensity distributions. We implemented the proposed framework using U-Net as the backbone model. The datasets were randomly divided into training, validation, and test sets in a ratio of 7:1.5:1.5. Data augmentation techniques, such as random rotation at small angles, horizontal flips, and random cropping, were applied to improve network robustness. Using the PyTorch framework, we trained our model with a batch size of 32 on four Nvidia TITAN RTX GPUs. The initial learning rate was set to 0.0001, and the number of communication rounds was 200. We used the Adam optimizer with $\beta$ set to (0.9, 0.999), and the hyperparameter $\mu$ was set to 1.0. The common metric Dice score was used to evaluate the model’s performance for both tasks.

## Results

This section presents a detailed comparison of the performance of the PCRFed framework with that of the state-of-the-art FL algorithms based on extensive experiments. Accuracy comparisons and ablation studies are thoroughly discussed.

### Accuracy comparison

First, we compared our method with several state-of-the-art FL frameworks: FedAvg [10], FedProx [16], FedBN [17], FedRep [35], and MOON [18]. The comparison results for the prostate MRI dataset and cervical cancer CT dataset are listed in Table 2. It is evident that FedAvg outperforms single-site training on average, highlighting the necessity of leveraging distributed data. FedProx achieved only minor improvements over FedAvg, indicating that the proximal term in FedProx had little impact on the training process. FedBN and FedRep exhibit inferior performance compared to the basic FedAvg, underscoring the advantages of tailored model personalization. Furthermore, we found that replacing the BN layer with an instance normalization IN layer effectively mitigates the retrogression problem caused by server aggregation in existing FL methods. Compared to other approaches, our framework demonstrates superior overall performance and higher single-client performance, achieving a 2.63% increase in the average Dice score for prostate segmentation.

**Table 2:** Comparison of state-of-the-art FL methods on prostate MRI images segmentation and cervical cancer CT image segmentation

Method	Prostate MRI (Dice %)							Cervical cancer CT (Dice %)								
	A	B	C	D	E	F	Average	1	2	3	4	5	6	7	8	Average
Single-site	89.34	87.43	92.01	81.58	83.44	85.23	86.51	65.43	50.77	60.75	64.45	48.75	54.39	56.52	69.89	58.87
FedAvg	90.67	89.06	92.61	82.40	84.24	86.84	87.64	70.05	55.92	65.89	69.39	73.75	60.08	61.30	68.26	65.58
FedProx	90.86	88.86	92.74	83.04	84.73	86.30	87.75	69.73	57.04	65.60	69.02	73.80	60.91	62.12	67.96	65.77
MOON	91.52	**89.93**	93.28	84.96	85.46	86.07	88.54	71.25	58.92	67.11	70.29	74.03	59.84	64.20	70.33	67.00
FedRep	90.47	89.41	93.19	83.45	87.07	86.83	88.40	70.73	59.21	69.39	70.15	73.04	61.30	63.26	69.42	67.06
FedBN	91.26	89.72	92.87	85.28	86.51	87.03	88.78	71.86	58.48	67.71	**71.06**	74.13	60.54	62.80	70.03	67.08
Ours	**92.03**	89.57	**94.29**	**87.54**	**88.24**	**87.58**	**89.88**	**72.51**	**59.83**	**70.06**	70.67	**76.02**	**63.54**	**64.33**	**71.86**	**68.60**

**Fig. 4:**
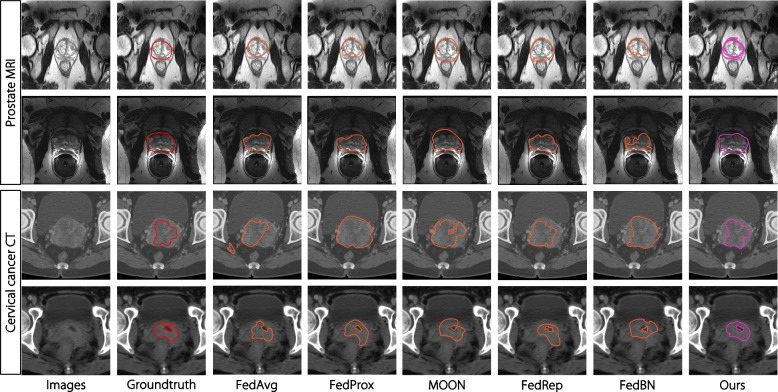
Visualization of segmentation results of different FL methods in prostate MRI (top two rows) and cervical cancer tumor CT image (bottom two rows) segmentation

In addition to performance enhancements, Fig. 4 presents a qualitative visualization of the segmentation outcomes compared to the baseline FL methods on the two segmentation datasets. Each row represents an image from a different client, illustrating dataset heterogeneity. In comparison to the ground truth, our approach precisely segments the anatomical structures, whereas other methods occasionally fail to capture the entire shape accurately.

### Ablation study

Ablation studies were conducted to investigate the key properties of the proposed PCRFed framework. To design the embedding layer, we experimented with various approaches, including adding a standard convolution layer or a depth-wise convolution layer. Additionally, to avoid increasing the number of parameters, we explored techniques such as average pooling and max pooling separately. Ultimately, we found that the best performance was achieved using an embedding layer that combined both max pooling and average pooling. This outcome may be attributed to the effective fusion of global and local information.

**Fig. 5:**
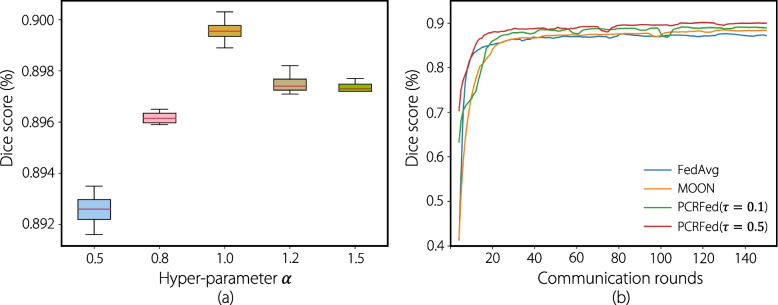
**a** Performance of PCRFed on a prostate MRI dataset with different hyper-parameter $\alpha$. **b** The average testing Dice score in different numbers of communication rounds on a prostate MRI dataset

To further assess the performance of our method, we analyzed the effects of different hyperparameters on the results. We varied the trade-off hyperparameter $\alpha = \{0.5, 0.8, 1.0, 1.2, 1.5\}$ in the prostate MRI dataset, as shown in Fig. 5a. Our findings indicate that a value of $\alpha =1.0$ generally achieves the best performance. We also examined the effect of the temperature parameter $\tau$. Figure 5b presents the average testing curves of the Dice score on prostate MRI using different FL methods. Our experimental results suggest that, for prostate MRI segmentation, the optimal value of $\tau$ is 0.5, whereas for cervical CT segmentation, $\tau =0.1$ yields the best performance.

Regarding the design of the adaptive parameter $\beta$, we investigated several normalization methods and found that exponential normalization outperformed linear and logarithmic normalization, particularly for clients with limited data. This suggests that exponential normalization more effectively guides the optimization process.

**Fig. 6:**
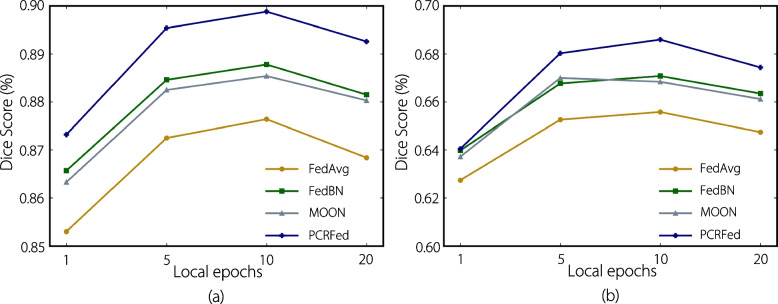
Comparison of the average Dice score with different numbers of local epochs for **a** prostate MRI and **b** cervical cancer tumor CT image segmentation

Finally, we explored the impact of local update epochs on our method, with results shown in Fig. 6. We observed that a smaller number of local epochs resulted in minimal model updates, leading to relatively lower accuracy within a fixed number of communication rounds. However, when the number of local epochs was too large, the accuracy of all methods decreased. Notably, our method outperformed all other FL methods across all cases, demonstrating its robustness and effectiveness.

## Discussion

Variations in image distribution and imaging operators across different sites and training-test sets pose significant challenges for applying FL methods. Traditional FL algorithms [10] assume that data are independently and identically distributed across participants, which does not hold for multisite medical imaging data. To address this challenge and optimize model prediction accuracy for each client, we propose a FL approach based on contrastive learning, which has proven effective in handling heterogeneity in multisite classification tasks [18]. By extracting latent representations, we capture site-specific heterogeneity and enhance the performance of U-Net-based models in medical image segmentation tasks.

The proposed weighted model-contrastive loss is derived from similarity constraints between local and global models. Several existing studies [16, 18, 41] have incorporated various regularization terms as similarity constraints. While sharing similar insights, our approach introduces a novel, lightweight implementation that enforces similarity constraints by blending local and global embeddings. Intuitively, the global embedding feature aids in learning general patterns, while the local embedding feature captures site-specific distributions. By combining these features, our method enables personalized models to fit individual distributions while preserving common knowledge.

Our FL framework prioritizes privacy preservation by securely storing private data on the client side, effectively mitigating the risk of raw data leakage. Ethical considerations are paramount in this study due to the sensitive nature of medical data and the privacy concerns associated with FL. By keeping sensitive data, such as patient MRI scans, decentralized, FL ensures that no data needs to be shared across institutions, enhancing privacy protection.

Additionally, the proposed approach minimizes communication costs when delivering aggregated models to clients. While local training requires retaining the previous local model, adding some computational overhead, this remains manageable within the medical FL setting, where hospitals prioritize improved diagnostic performance. Our FL algorithm is computationally efficient, utilizing four Nvidia TITAN RTX GPUs-consumer-grade hardware-ensuring minimal overhead. This makes deployment scalable across multiple medical centers while maintaining efficiency and ease of integration into existing infrastructure.

The PCRFed framework shows great potential, but several limitations must be addressed in future work. A major challenge is data heterogeneity across medical institutions, including variations in imaging protocols, machine types, and patient demographics. These differences can cause domain shifts, reducing the model’s ability to generalize. To enhance performance, more robust domain adaptation techniques are needed. Another key challenge is the framework’s reliance on high-quality expert annotations, which are scarce and expensive in the medical field. Exploring semi-supervised or weakly supervised learning approaches could help alleviate this issue. Additionally, the computational complexity of integrating contrastive learning with FL may limit scalability, particularly in resource-constrained settings. Optimizing these methods is crucial for broader deployment. Personalized learning in FL also faces the risk of overfitting, especially with small datasets. Advanced meta-learning approaches could help mitigate this issue. Although federated learning enhances privacy by keeping data local, the framework has yet to be fully evaluated under various privacy-preserving mechanisms, making this an important area for future research. Furthermore, we plan to investigate the role of parallel computing in improving performance. Leveraging parallel processing on both client devices and the central server can help address client heterogeneity, reduce computational and communication bottlenecks, and improve system scalability. Another critical challenge is the impact of noise and artifacts in medical images, which can degrade model performance [42]. A potential solution is integrating super-resolution (SR) techniques to reduce noise and enhance image quality, allowing the model to better capture relevant features for segmentation tasks. Incorporating SR-based approaches into FL frameworks, particularly in multicenter environments, presents an exciting direction for future research.

In general, our PCRFed approach is agnostic to the model architecture and can potentially be combined with other FL techniques such as frequency-domain transformation [21, 36] and aggregation algorithms [19, 20]. In the future, we will focus on exploring more versatile and efficient algorithmic frameworks.

## Conclusions

This study introduces a novel FL framework called PCRFed, which is designed to address non-IID challenges in medical scenarios. To enhance the performance of federated deep learning models in segmentation tasks, we introduced a personalized paradigm that utilizes contrastive learning methods to adaptively optimize local clients with global information. The efficacy of this framework was thoroughly demonstrated through experiments conducted on two medical image segmentation datasets, and our method holds significant potential for advancing the impact of FL in real-world medical applications.

## Data Availability

The prostate MRI image datasets analyzed in this study can be found in the ‘A Multi-site Dataset for Prostate MRI Segmentation’ repository, https://liuquande.github.io/SAML/. The cervical CT image datasets analyzed during the current study are not publicly available for the protection of patient privacy but are available from the corresponding author on reasonable request.

## Abbreviations

MRI: Magnetic resonance imaging
CT: Computed tomography
FL: Federated learning
non-IID: Non-independently and identically distributed
PFL: Personalized federated learning
NT-Xent: Normalized temperature-scaled cross-entropy
BCE loss: Binary cross-entropy loss
ROI: Region of interest
BN: Batch normalization
SR: Super-resolution
